# Nanomaterial application for protein delivery in bone regeneration therapy

**DOI:** 10.1590/1414-431X2024e14057

**Published:** 2025-02-03

**Authors:** B.S. Hariawan, A. Miatmoko, Q.K. Anjani, F. Annuryanti, D.B. Kamadjaja, A. Nurkanto, Purwati, D.M. Hariyadi

**Affiliations:** 1 Master Program of Pharmaceutical Sciences, Faculty of Pharmacy, Universitas Airlangga, Campus C UNAIR Mulyorejo, Surabaya, Indonesia; 2 Department of Pharmaceutical Sciences, Faculty of Pharmacy, Universitas Airlangga, Campus C UNAIR Mulyorejo, Surabaya, Indonesia; 3 Stem Cell Research and Development Center, Universitas Airlangga, Campus C UNAIR Mulyorejo, Surabaya, Indonesia; 4 Pharmaceutics and Delivery System for Drugs, Cosmetics and Nanomedicines Research Group, Faculty of Pharmacy, Universitas Airlangga, Campus C UNAIR Mulyorejo, Surabaya, Indonesia; 5 Skin and Cosmetics Technology Centre of Excellent, Faculty of Pharmacy, Universitas Airlangga, Campus C UNAIR Mulyorejo, Surabaya, Indonesia; 6 Medical Biology Centre, School of Pharmacy, Queen's University Belfast, Belfast, Northern Ireland, UK; 7 Department of Oral and Maxillofacial Surgery, Faculty of Dental Medicine, Universitas Airlangga, Surabaya, Indonesia; 8 Research Center for Biosystematics and Evolution, Research Organization of Life Sciences and Environment, National Research and Innovation Agency, InaCC Building Soekarno Science and Technology Area, Cibinong, Indonesia

**Keywords:** Disease burden, Bone regeneration, Nanotechnology, Scaffold

## Abstract

Bone fractures must undergo a complex healing process involving intricate cellular and molecular mechanisms. They require a suitable biological environment to restore skeletal stability and resolve inflammation. Scaffolds play a vital role in bone regeneration, thus reducing disease burden. Autologous bone graft represents the gold standard of therapy. However, its application is limited due to various reasons. Nanotechnology, in the form of nanomaterials and nano-drug delivery systems, has been proven to increase the potency of active substances in mimicking extracellular matrix (ECM), thereby providing physical support benefits and enhancing therapeutic effectiveness. Various materials, including protein, metal oxide, hydroxyapatite, and silica are modified with nanoparticle technology for the purposes of tissue regeneration therapy. Moreover, the properties of nanomaterials such as size, seta potential, and surface properties will affect their effectiveness in bone regeneration therapy. This review provides insights that deepen the knowledge of the manufacturing and application of nanomaterials as a therapeutic agent for bone regeneration.

## Introduction

Multiple factors are involved in bone healing and new bone formation, and they act via various cellular and molecular processes. Self-healing occurs independently of medical treatment and is controlled by numerous systemic and local factors (1). In certain cases, including atrophic non-union, osteomyelitis, osteosarcoma, osteomalacia, osteoporosis, and avascular necrosis, the self-healing abilities are not able to fully repair bone fractures (2). Therefore, surgery and additional bone fracture involving scaffold allografts and autologous bone grafts are necessary.

Scaffolds play a vital role in bone regeneration by triggering mesenchymal stem cells to differentiate into bone cells. Consequently, the scaffold must contain biocompatible biomaterials and have osteointegration and osteoinductive properties (3). Autologous bone grafts, the gold standard in bone regeneration therapy, have all those properties while involving negligible risk of immunological rejection because it is obtained from the patient him/herself. However, clinical application of autologous bone graft is limited since the amount of bone graft available varies from one patient to another. In addition, performing multiple grafts exacerbates the risk of complications such as infection, prolonged wound drainage, large hematoma, and re-operation (4). Therefore, a new alternative for scaffold material is necessary for tissue regeneration therapy.

The scaffold is also intended to be a medium that will interact with surrounding cells and tissues to improve or accelerate the bone healing process. The scaffold is modified in such ways as to mimic the biological environment of bone tissues. Human bones are composed of a combination of diverse biological materials and demonstrate high mechanical strength. These components form a complex structural architecture from the nanoscale to the macroscale (5). Metal ions, such as Zn, can be combined with hydroxyapatite (HA) scaffolds or polymers with nanoparticle delivery systems to effectively mimic the bone mineralization process, thereby enhancing osteoinduction and osteointegration (6).

Various growth factors and biologically active ingredients such as transforming growth factor (TGF), bone morphogenetic protein (BMP), and insulin-like growth factor (IGF) are delivered by nanocarrier delivery systems to resemble the extracellular matrix (ECM) (7). The ECM consists of polysaccharides and proteins secreted and regulated by cells to modulate cell activity (7,8). One benefit of nanocarriers in the delivery of growth factors and other biologically active substances is their ability to provide a controlled release in areas of damage, thereby enabling cell migration processes to occur (9,10). In the regeneration process, the migration of cells to the damaged area resulting in angiogenesis, fibrosis, chemokine secretion, and stem cell differentiation is crucial. Therefore, various means of increasing the effectiveness of these processes have been investigated. This review will discuss the use of various nanocarriers in bone regeneration therapy.

## Bone healing

### Type of bone defect

Bone fractures are categorized into various types according to specific criteria. Regarding the post-fracture displacement of bones, fractures are divided into displaced and non-displaced varieties. Fractures that are not accompanied by bone displacement are generally treated by a non-operative treatment (11). Displaced fractures, which occur when a bone moves from its original position, result in more severe damage and require a more complicated therapy. For example, tearing the ascending cervical branch that blocks the supply of the arterial ring formed by the circumflex artery is necessary in the case of a displaced fracture of the femoral neck, which can impact the existing blood supply. This treatment can compromise the healing ability of fractures and lead to non-union or osteonecrosis (12).

Bone defects have various causes, including trauma, congenital anomalies, and tissue resection due to cancer (13). Bone defects are distinguished by their size, and critical bone defects, almost invariably, require replacement therapy (14). Critical bone defects are those greater than 1-2 cm and the loss of bone circumference exceeds 50% due to high-energy trauma to soft tissue and periosteal stripping, especially in blast injuries, high-grade open tibial fractures, tumor resection, and infections requiring extensive debridement (15). However, such defects are influenced by their anatomical location and the condition of the surrounding soft tissue (15).

### Bone tissue regeneration

**Figure 1:**
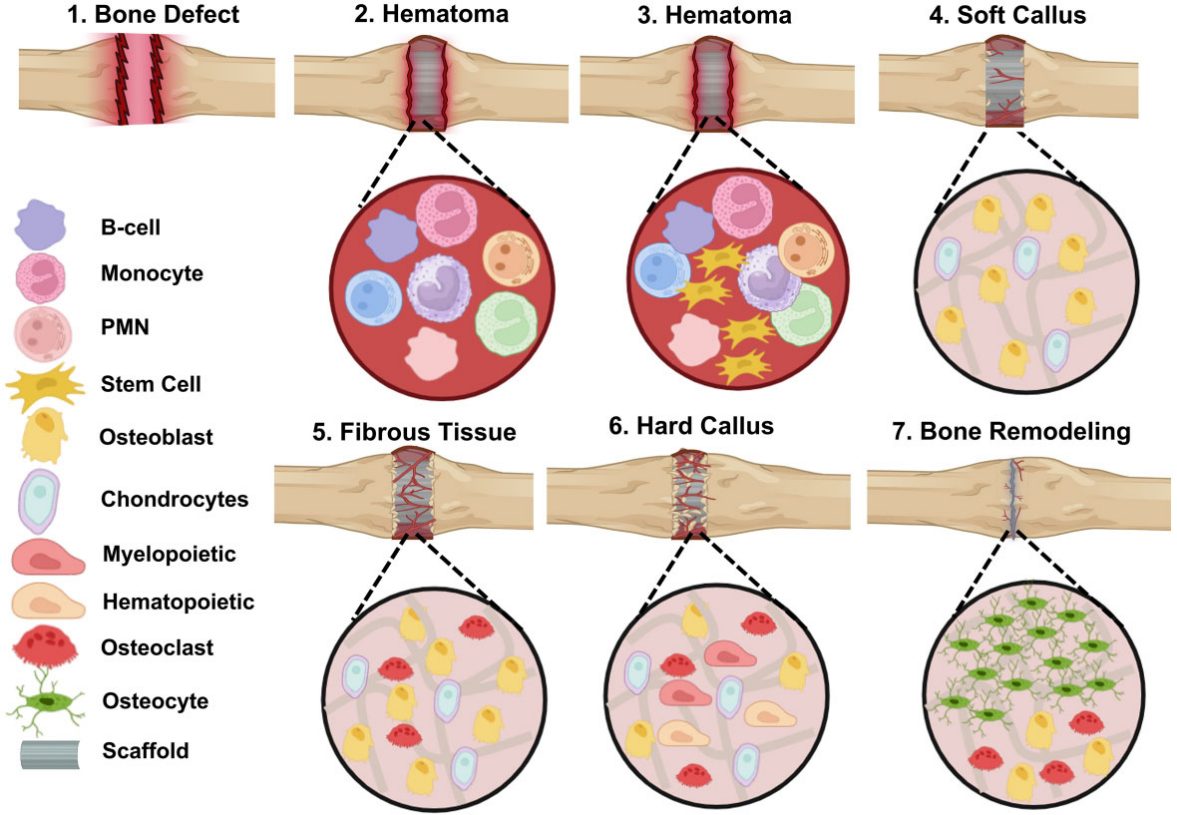
The hematoma phase of the bone repair process is followed by the arrival of mesenchymal stem cells (MSCs), which later differentiate into osteoblasts during the soft callus phase. This is when blood vessels start to form. Following that, fibrous tissue starts to develop, and osteoclast cells start to emerge. After the formation of a hard callus and the appearance of hematopoietic cells, osteoblast cells eventually develop into osteocytes, resulting in bone remodeling. PMN: polymorphonuclear cells.

Broken bones heal after trauma either directly through bone fusion or indirectly through the formation of calluses, as shown in Figure 1. Intramembranous and endochondral ossification constitute two common indirect healing mechanisms in bone regeneration (16) that are also known to be basic mechanisms of the bone formation process. Intramembranous ossification can take place following direct bone deposition onto the surface during bone remodeling. It entails the direct differentiation of progenitor cells into osteoblasts, which produce bone (17). When primary bone healing occurs without the use of intermediate secondary cartilage, intramembranous bone formation represents the primary mechanism. This process can also occur during bone regeneration (18), which is also referred to as primary bone healing (16). Direct bone union, or primary bone healing, takes place in tiny fracture gaps, typically between 10 and 100 μm in size, with less than 2% strains. For the fracture to remain completely stable during this process, rigid fixation similar to traditional compression plates is frequently essential.

According to Stewart et al. (18), the second mechanism involves the formation of endochondral bone, which is responsible for most fracture calluses, longitudinal bone growth at the physis, and long bone growth in embryos. Mesenchymal cells differentiate to form cartilage during early bone formation, which is subsequently invaded by blood vessels. When osteoblasts form bone on a cartilage template that has been partially destroyed by clastic cell activity, blood vessels act as a transit pathway for the cells. Stewart et al. (18) state that during long bone growth, endochondral ossification takes place at the growth plate (physis). Enhancing the flexibility of fixation systems such as locking plates can facilitate secondary healing, which is more prevalent in fissure fractures with larger micromovements. In intramembranous ossification, the bone tissue is directly synthesized by the bone marrow, while in endochondral ossification, cartilage forms and calcifies (16).

A hematoma forms around the fracture site due to the rupturing of the periosteum and blood vessels that supply the bone. The hematoma subsequently clots, creating a makeshift framework that aids in subsequent healing. Hematoma formation serves as a source of inflammatory cells that release growth factors and pro-inflammatory cytokines, including BMP, interleukins (IL-1, IL-6, IL-11, and IL-23), and tumor necrosis factor-alpha (TNF-α) (19). Cells are drawn to the damaged tissue following the release of growth factors and pro-inflammatory cytokines. Inflammation begins immediately after cells migrate to the damaged bone site, thereby initiating the regeneration process.

The next stage is the formation of a fibrocartilaginous callus between day 5 and day 11 (19). After the process of cell migration, secretion of vascular endothelial growth factor (VEGF) ensues. VEGF is a key regulator of vascular growth processes required for the effective coupling of angiogenesis and osteogenesis during bone development and repair (20). Angiogenesis is triggered at the site by the release of VEGF, after which fibrin-rich granulation tissue then begins to form inside the hematoma. Endochondral ossification of the cartilage callus occurs from day 11 to day 28. Further differentiation of chondroblasts, chondroclasts, osteoblasts, and osteoclasts is stimulated by the expression of receptor activator of nuclear factor kappa-beta ligand (RANK-L). Consequently, the cartilage of the callus is absorbed and starts to calcify. The final stage of bone healing is the bone remodeling process. This stage occurs from day 18 and can last from months to years (19).

### Osteoblast differentiation

**Figure 2:**
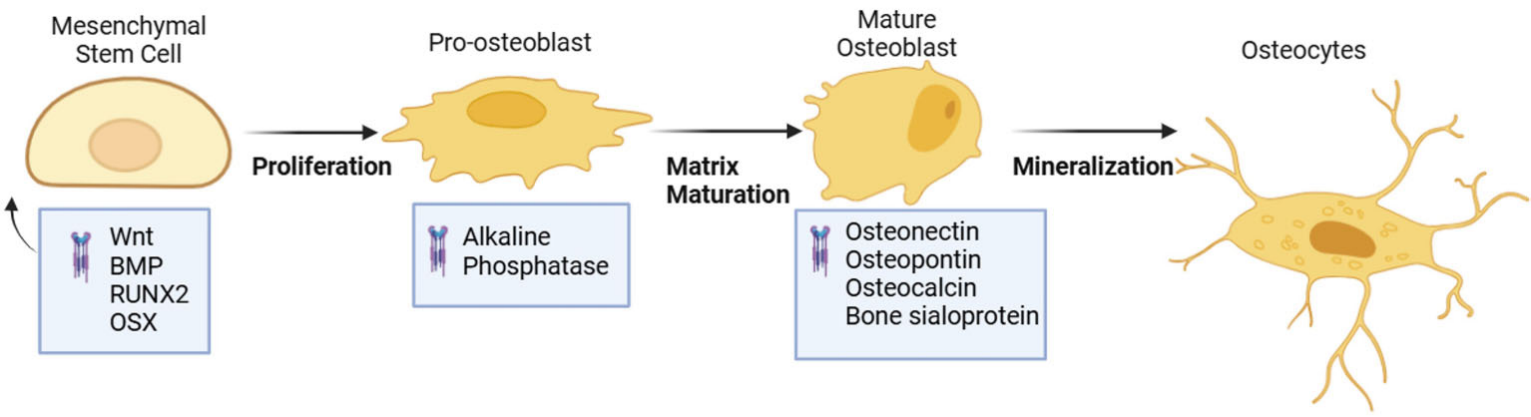
Mesenchymal stem cells (MSCs) undergo a differentiation process that involves the activation of osteogenic transcription factors Wnt, Osterix (OSX), and Runx-2. The cells proliferate and express collagen, bone morphogenetic protein (BMP), and transforming growth factor (TGF-β1). The cell then exits the cell cycle and undergoes differentiation, expressing the extracellular matrix's collagen and alkaline phosphatase. Matrix maturation is associated with increased expression of alkaline phosphatase and other non-collagenous proteins, including osteocalcin, osteopontin, and bone sialoprotein. When osteocalcin is exposed to the scaffold, matrix mineralization occurs, and the deposition of mineral materials begins, resulting in osteocytes.

Bone is a tissue composed primarily of osteoblasts. Osteoblasts are cells derived from mesenchymal stem cells (MSCs) through a differentiation process regulated by canonical Wnt/β-catenin signaling pathways (21). During this differentiation process, Runx-2 and osterix, osteogenic transcription factors, will experience increased expression, while that of transcription factors C/EBPα and PPARγ will decrease (22). Sox9 acts prior to Runx2 activation in the endochondral pathway, preventing stem cells from differentiating into osteoblasts and chondroblasts. As demonstrated in Figure 2, once Runx2 is activated cells characterized as preosteoblasts undergo three stages of differentiation: proliferation, matrix maturation, and mineralization (21).

Cells multiply and express TGFb1 receptors, collagen, osteopontin, fibronectin, and collagen during stage 1 of osteoblast differentiation. In stage 2, they emerge from the cell cycle and differentiate, bringing collagen and Alp in the extracellular matrix to maturity. The osterix transcription factor is necessary for differentiation into mature cuboidal osteoblasts that actively mineralize bone matrix (22). However, before the freshly laid matrix can become mineralized, it needs to mature. Increased expression of alkaline phosphatase and a number of non-collagenous proteins, such as osteocalcin, osteopontin, and bone sialoprotein, are linked to matrix maturation (22). When osteocalcin is added to the organic scaffold, matrix mineralization takes place in stage 3 and initiates the deposition of mineral materials. After collagen, osteocalcin is the second most prevalent protein in bones. At this point, the osteoblast is cubic in shape.

## Protein for bone regeneration therapy

### Transforming growth factor beta (TGF-**β**)

TGF-β is a multifunctional growth factor homodimer measuring 25 kDa with a range of biological activities in various cell types and tissues (23). One of the triggers for the formation of TGF-β are platelets in the blood clot that forms after the fracture. One of the largest producers are fibroblast cells. Once synthesized, TGF-β forms a complex with latency-associated peptides and is stored in the extracellular bone matrix (23).

In the bone regeneration process, TGF-β plays a role in both cell proliferation and cell differentiation processes. TGF-β also plays a role in extracellular matrix synthesis (23). In terms of future clinical applications, TGF-β has been studied for its benefits as an agent with osteoinductive properties and is one of the clinical biomarkers used to detect the occurrence of this process.

One of the studies showing the benefits of TGF-β in tissue regeneration was conducted by Zhang et al. (7). In this study, CCK-8 and flow cytometry assays carried out on osteoblast cells confirmed an increase in cell proliferation after additional treatment with TGF-β. Intensified osteogenic activity was also observed by means of Alizarin Red staining after TGF-β treatment, characterized by an increase in the biological marker ALP, as well as a proliferation of mineralization markers and levels of Ca_2_+ deposition.

### Fibroblast growth factor (FGF)

FGF is a growth factor produced by numerous cells, one type of which are osteoblasts. FGF measuring 17-34 kDa consisting of 22 ligands is stored in an active form in the extracellular bone matrix. FGF secretion by cells can trigger internal changes and changes in the communication between cells, thus inducing other changes in surrounding cells (23,24). When binding to FGFR, FGF can activate three downstream pathways, namely the Ras/MEK/MAPK/ERK, the PI3K/AKT, and the PLCγ pathways, each of which function within the cell. PLCγ pathways will affect the cytoskeleton, while the PI3K/AKT pathway will influence cell survival, and the Ras/MEK/MAPK/ERK pathway will influence the processes of cell proliferation and differentiation (25). Apart from these valuable functions, FGF is also known to play an important role in the processes of angiogenesis and cell migration (23).

The use of FGF as a clinical growth factor therapy for tissue regeneration has previously been studied. For example, Kawaguchi et al. (26) conducted research into the effect of rhFGF doses of 200, 400, and 800 μg with a gelatin hydrogel carrier in the osteotomy healing process with the parameters being the percentage of patients with radiographic bone union and the time required for union formation. The results showed that large doses of rhFGF-2 (800 μg) resulted in the highest percentage of patients with radiographic bone union and reduced the lead time to union formation. In addition, a randomized, placebo-controlled trial conducted on tibial fracture patients indicated that hydrogen gelatin injection containing rhFGF-2 increased the percentage of patients with radiographic bone union compared to the placebo group without rhFGF-2.

### Insulin growth factor (IGF)

Another growth factor executing an important role in the tissue regeneration process is IGF. There are at least two isomers of IGF: IGF1 and IGF2. Insulin-like growth factor-1 (IGF-1) is a 70 amino-acid single-chain peptide with a molecular weight of 7.6 kDa. During the process of cell differentiation, IGF will bind to the IGF-1R receptor to produce insulin receptor-substrates (IRS). After phosphorylation, IRS will activate PI3K and extracellular signal-regulated kinase (ERK)/ERK, which is one of the mitogen-activated protein kinase (MAPK) cascade members (27). This will, in turn, activate mTOR with the result that it can induce chondrocyte proliferation and differentiation (27).

A study by Wang et al. (28) revealed the effect of IGF1 on the proliferation and differentiation of stem cells from apical papilla (SCAPs), cells responsible for root pulp and dentine formation. The results show that the administration of 100 ng/mL IGF-1 increased SCAP proliferation and differentiation as indicated by an increase in the ALP marker. The mineralization capacity of SCAPs, which was examined using alizarin red staining, also increased. Furthermore, western blot and RT-PCR analysis showed that the expression of osteogenic proteins and related genes (ALP, RUNX2, osterix, and osteocalcin) was significantly increased (28).

### Platelet-derived growth factor (PDGF)

PDGF is one of the main regulators of tissue regeneration. PDGF, a 30-kDa dimeric protein composed of disulfide-linked polypeptides (23), has three isoforms: PDGF-AA, PDGF-BB, and PDGF-AB. During a trauma process, in addition to VEGF, PDGF plays a role in the activation and migration of inflammatory factors such as neutrophils (29). Moreover, PDGF also has an important role, especially regarding the production and synthesis of other growth factors essential for tissue regeneration and the healing process. Other findings show that PDGF is vital to the tissue regeneration process through activation of ERK 1/2 signaling pathway resulting in osteoblast differentiation (30).

In the osteoblast differentiation process, several master regulators, namely ATF4, RUNX2, and β-catenin, are activated by ERK 1/2 (31). After activation of these master regulators, MSC cells differentiate into preosteoblasts and osteoblasts. The latter undergo endochondral ossification and intramembrane ossification processes culminating in complete bone healing (32).

### Vascular endothelial growth factor (VEGF)

The dimeric protein known as VEGF-A, which is the biologically active version of VEGF, is composed of several variations (121-206 amino acids) of a single gene. The majority of cell types create numerous VEGF forms simultaneously. When hypoxia or other cytokines are present, their expression increases. Angiogenesis and capillary permeabilization are both induced by VEGF. New blood vessels develop during bone regeneration, which are vascular alterations that are critical for the delivery of nutrients, the movement of macromolecules, and the invasion of cells (23).

Acute inflammation sets in shortly after bone trauma and a hematoma composed of neutrophils and red blood cells forms at the injury site. Following bone damage, VEGF is concentrated in the hematoma because hypoxia triggers VEGF expression and the hematoma's fibrin matrix may act as a VEGF reservoir. Because inflammatory stimuli decrease neutrophil retention in the bone marrow and circulating neutrophils are drawn into the hematoma to clear away microbiological pathogens and bone debris, neutrophils in the peripheral circulation proliferate immediately after injury. Furthermore, it is known that VEGF increases the permeability of sinusoids in the bone marrow and triggers neutrophil chemotaxis (33).

## Drug delivery systems for bone regeneration

### Liposomes

**Figure 3:**
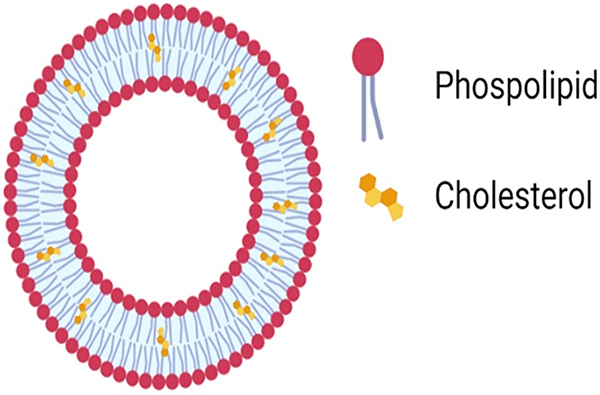
Structure of a liposome.

As shown in Figure 3, liposomes are sphere-shaped vesicles consisting of one or more phospholipid bilayers (34). Liposome properties differ considerably with lipid composition, surface charge, size, and the method of preparation (34). Liposomes are nanocarriers largely used even in the clinical stage because their composition is biodegradable, biocompatible, non-toxic, and non-immunogenic (35). The amphiphilic phospholipid bilayer of liposomes closely resembles mammalian cell membranes, allowing efficient interaction between liposomes and cell membranes and subsequent effective cellular uptake (36).

As a drug delivery system, liposomes can protect encapsulated substances from physiological degradation, extend drug half-life, and control the release of drug molecules while also demonstrating excellent biocompatibility and security (37). Furthermore, liposomes can be augmented with ligands to increase efficiency and specifically target damaged cells, thereby enhancing the pharmacokinetics of liposomes and their ability to cross target membranes, achieving high concentrations within cells while reducing toxicity and increasing treatment efficacy (35). The encapsulation efficiency of liposome hydrophilic compounds improves with liposome size and decreases with the number of bilayers.

Based on size and number of bilayers, liposome structures are divided into four groups: small unilamellar vesicles (SUV), large unilamellar vesicles (LUV), multilamellar vesicles (MLV), and multivesicular vesicles (MVV) (34). They are made up of one or more lipid bilayers that enclose watery sections. They may range from tens of nanometers to tens of microns in diameter. They can be formed from a great variety of lipid constituents, leading to a wide range of physical properties and allowing manipulation of their properties. Hydrophobic drugs are incorporated into the lipid bilayers, while hydrophilic drugs are usually encapsulated in the aqueous compartments. In MLV liposomes, the area of the hydrophilic core decreases with the increasing number of bilayers. Because of that, the drug loaded into the liposome decreases (34).

### Exosomes

Exosomes are single membrane vesicles secreted from cells that can be used to perform physiological functions (38). Exosomes are also referred to as 40-100-nm vesicles released during reticulocyte differentiation due to the fusion of multivesicular endosomes with the plasma membrane (38). As a result of excretion from the cell, the single-membrane exosome possesses the same topology as the cell.

Cell topology refers to the arrangement and connectivity of cells within a tissue or structure (39). Exosomes are derived from cells and formed of lipid bilayers, similar to cell membranes (40). They are essential mediators of intercellular communication and regulators of the cellular niche. Many diseases can change the characteristic of cell topology. This finding leads to the idea of using exosomes as drug delivery vehicles, especially for gene therapy (41). Exosomes are now regarded as a distinct cellular entity capable of carrying cargo of cell components (RNA and protein) to be shared between cells.

Exosomes are heterogeneous, with sizes ∼30 to ∼200 nm in diameter, they vary significantly, even for ones secreted from a single cell line (42). Apart from size, exosomes also have a heterogeneous composition. They are enriched with membrane-bound oligomeric protein complexes and are rich in tetraspanins, adhesion molecules, enzymes, scaffolds, RNA-binding proteins, RNA, DNA, and complex glycans (42).

The properties of the exosome are directly related to how it is purified. Exosomes and microvesicles were traditionally purified by differential centrifugation, involving pelleting of the microvesicles at 1-2 rounds of 10,000 *g* for 30 min, whereas exosomes were pelleted by rotating the supernatant 10,000 *g* rounds for 90-120 min. However, this exosome pellet fraction contains other components besides exosomes. If the starting material is a biofluid such as plasma, these pellets may contain large amounts of chylomicrons and other lipoprotein particles, especially if the subject has recently consumed food (42).

Exosomes mediate communication between cells, one form being the transfer of proteins, gene components, and lipids from donor cells to recipient cells (43). Based on this function, the exosome, which already acts as a natural delivery system, has inspired its development as a modern delivery system. As a drug delivery system, exosomes easily penetrate body tissues, diffuse into the blood, and even cross the blood-brain barrier (44). In addition, exosome-mediated delivery through the P-glycoprotein drug efflux system may reduce drug resistance. Compared to synthetic drug carriers, exosomes isolated from patients' cells have higher biocompatibility and lower toxicity. Exosomes can directly excite target cells and transfer membrane receptors between cells, thus avoiding diffusion into tissues by phagocytes/monocytes.

Exosomes have become one of the selected techniques for protein delivery systems (45,46). Several protein delivery methods mediated by lipid nanoparticles, such as liposomes and lipid-based nanocarriers, have drawbacks related to the absence of a separation mechanism between cargo proteins and lipid nanoparticles. This particular lack means not only that they limit the efficiency of cytosolic delivery, but also that the preparation of these particles often involves complicated protein purification stages (47). Protein loading/trapping with the exosome can be carried out by endogenous biogenesis processes. In contrast, the release process is assisted by surface modifications on the receptors that can trigger release from the exosome (47).

Recombinant proteins can be passively loaded into exosomes *in vitro* using various techniques, including sonication, freeze-thaw, extrusion, and simple incubation. In the endogenous passive loading by protein overexpression, exosomes passively load proteins, causing cells to overexpress target proteins in low-probability ways. Proteins can bind to the inner layer of the exosomal membrane using the exosomal membrane-anchored protein technique, which allows them to conjugate with exosomes during the process of natural exosome formation. Using optogenetically reversible protein-protein interaction to load proteins into exosomes, proteins are actively loaded into exosomes under light. Because the interaction is reversible, the proteins in the exosome lumen separate as free forms from the exosome membrane (48).

Clinical trials of exosome use have been conducted in various diseases. Nonetheless, there are evaluation-related difficulties primarily because determining the uniqueness and purity of exosomes is necessary to assess their efficacy and safety. Due to the low yield during the extraction and isolation stage of exosomes, several sources are required (49) along with scalable isolation methodologies and repeatable procedures (50). Currently, in isolation based on size and density, ultracentrifugation is the gold standard method because it is easy and does not require various reagents (50). The high purity of exosomes is important, especially related to the safety of the preparation. In this regard, the International Society for Extracellular Vesicles (ISEV) issued a statement regarding several characteristics of the process and products that should be considered when working with exosomes (51).

### Micelles

Micelles are spherical structures approximately 10-100 nm in size composed of amphiphilic surfactants. Micelles have a hydrophobic core and a hydrophilic shell to deliver hydrophilic active ingredients, which dissolve in water (52). An amphiphilic molecule dissolved in a solution is in the form of a surfactant. At a certain concentration, after passing through a critical micelle concentration (CMC), this unimer undergoes self-aggregation to form a core-shell structure called a micelle. One type often used in research is polymeric micelles, which can form nano-sized stable micelles with modifiable surface characteristics (53).

Besides their application in bone regeneration therapy, polymeric micelles are also used to modify drug release. The modification process is carried out using various lengths of polymer blocks (53). Moreover, polymeric micelles are employed because they can be effectively internalized by cells. One active ingredient for osteoinduction, dexamethasone, has cell receptors and therefore must pass through cell membrane barriers. The mechanism of micelle internalization into the cell is that of clathrin endocytosis (54). Research has shown that surface modification of micelles affects the mechanism of cell internalization. Micelles with surface polyethylene glycol affect integrin targeting specificity, resulting in more extensive cell uptake (54). The characteristics of micelles and their ability to modify themselves render them as potential nanocarriers in tissue regeneration therapy.

### Microspheres

Microspheres are tiny spherical particles with a particle size ranging from 1 to 1000 micrometers. Microspheres are freely movable protein- or polymer-based microparticles (55). There are two types of microspheres: micromatrices and microcapsules. Micromatrices containing the encapsulated substance spread throughout the matrix, whereas microcapsules are small containers with capsule walls surrounding their internal contents. Microspheres are drug particles dispersed at a molecular or macro scale in a matrix composed of one or more soluble polymers. They are described as “monolithic spheres or therapeutic substances dispersed either as a dispersion of molecular particles or the entire matrix” (55).

The selection of the polymers will determine the biodegradability, toxicity, and characteristics of the microsphere. Synthetic or natural polymers degraded *in vivo* that can produce biocompatible or non-toxic by-products and progressively release dissolved or dispersed drugs are suitable (56). Polyethylene and polystyrene are the two most frequently used types of polymer in microspheres. Polystyrene represents a suitable choice for biomedical delivery because of its capacity for antibody deposition. Polystyrene can strongly bind proteins and ligands and is, thus, useful for biological product delivery (55). Drug release can be modified and delayed through microencapsulation (57).

### Hydroxyapatite

Bone is an organ formed of two components, a mineral phase and an organic phase, consisting of collagen and non-collagen proteins, respectively. The mineral in bone, calcium phosphate, has been widely used as a biomaterial in tissue regeneration therapy (58). Calcium phosphate (CaP) has osteoconductive and osteoinductive properties. The release of calcium and phosphorus ions regulates osteoblast and osteoclast activation, thereby facilitating bone regeneration (58). CaP biomaterials are available in various compositions, including hydroxyapatite, beta-tricalcium phosphate, and biphasic CaP. In the terms of physical form, CaP biomaterials can be found in particulates, blocks, cement, coatings on metal implants, and composites with polymers. Of the several varieties, hydroxyapatite (HA or HAP) and β-tricalcium phosphate (β-TCP) are clinically approved for orthopedic and maxillofacial surgery (59).

HA is a bone-building material that fills nearly 70% of the bone. CaP induces tissue regeneration by activating the RANKL in osteoblasts, thereby inducing osteoclast differentiation (60). HA demonstrates good biocompatibility properties and is easily modified into a scaffold with specific porosity and size (up to the nanoscale), rendering it suitable for the osteogenesis process (58). Various studies examined how the characteristics of HA affect osteogenesis (61). For example, Davison et al. (61) demonstrated that β-TCP with features of submicron structure facilitates more effective differentiation of human peripheral blood monocytes into osteoclasts compared to that of the microstructure. Another study demonstrated that the surface structure of the BCP influences osteoclastogenesis and bone formation after implantation into the muscles of dogs (62).

### Silica

Mesoporous silica nanomaterials (MSNs) have been developed as an alternative in tissue regeneration therapy due to their low toxicity and good biocompatibility (63), high porosity (64), and strong mechanical properties (65). In addition, Si ions released during MSN degradation can increase the expression of osteogenesis-related genes (OCN, RUNX2, and OPN) in osteoblasts to promote bone repair processes (66). The biological activity and biocompatibility of MSNs are closely related to their crystallinity. The number of siloxane rings in the MSNs structure will determine its crystallinity (67). MSN, which has a low crystal structure, contains a small number of siloxane rings. Low crystal structure means lack of crystallinity. The degree of structural order shown by the molecules within a polymer is measured by the so-called crystallinity (68). Amorphous polymers are defined as those having negligible crystallinity (69). Crystallinity is inversely correlated with the polymer's permeability. It has also been shown that a higher capacity for water absorption is associated with a lower crystallinity index (68). The naturally low toxicity of silica-based mesoporous nanomaterials sets them apart from a variety of other inorganic nanomaterials, including other silica-based nanomaterial forms like nanoquartz and fumed silica, which are linked to high surface reactivity and poor biocompatibility. The strained 2- or 3-member siloxane rings are commonly found in high-temperature silica types, such as quartz and fumed silica, and they can be harmful because surface reconstruction opens these rings to display vicinal silanols (70).

The mesoporous formation also lowers its crystallinity, which results in improved biocompatibility (70). Other studies have shown that the biocompatibility of MSN is affected by silanol groups in the outer layer that interact negatively with biological molecules and destroy their structure (71). The cationic charge on the surface can produce an immune reaction and cytotoxicity that is relatively significant in contrast to neutrals and anions (71).

MSN with a high specific surface area is widely used as a nanocarrier to load drugs and large macromolecules such as proteins to obtain controlled release (72). Research by Liang et al. (73) shows that immune-mediated osteogenesis may be induced by the application of MSN, which can trigger the formation of a suitable immune environment and induce macrophage uptake of nanomaterials.

### Metal oxides

Cerium oxide nanoparticles (CeO_2_ NPs), iron oxide nanoparticles (IONPs), and zinc oxide nanoparticles (ZnO NPs) are types of metal oxide nanoparticles widely used in tissue regeneration therapy (74,75). Each type of metal oxide nanomaterial has different characteristics and advantages. ZnO NPs have good biocompatibility and antibacterial properties. In tissue regeneration therapy, applying ZnO NPs into biomaterials such as chitosan, alginate, and hydrogel, can help induce tissue integration of these materials by promoting fibroblast integration, neo-angiogenesis, and wound healing (76). Neo-angiogenesis induction activity is thought to be related to ZnO NPs, which can produce zinc ions and induce the formation of reactive oxygen species (ROS) (77). ROS play a vital role in angiogenesis as a starting point for cell proliferation and migration.

During tissue regeneration therapy, IONPs are used as labeling agents in non-invasive monitoring of *in vivo* therapy. Research by Fayol et al. (78), who used IONPs for MSC cell labeling in the processes of chondrogenic differentiation and proliferation of human bone marrow (BM)-MSCs, demonstrated that chondrogenic genes downregulation was time-dependent and that IONPs were relatively stable in biochemical media at concentrations suitable for use in non-invasive monitoring. This discovery was made after the previous use of metal oxide labeling was found to have certain drawbacks, primarily related to nanoparticle instability prior to cell uptake causing misleading MRI cell tracking and impaired cell differentiation (78).

Other studies have shown that magnetic stimulation can be used to create controlled drug release systems within polymeric delivery systems. Previous studies focusing on IONPs encapsulated from chitosan and heparin biopolymer nano gels for BMP-2 vector delivery showed that IONPs could stimulate BMP-2 release in a controlled manner and demonstrated efficiency in promoting MG-63 cell viability (76). Through the use of an applied magnetic field, the release of transforming growth factor beta (TGF-β)1 with a heparin-binding domain is achieved in a controlled manner (79).

Another metal oxide nanoparticle, CeO_2_ NPs, can enhance the healing process of various tissues due to its excellent biological properties, including anti-oxidation, anti-inflammatory, antibacterial activity, and angiogenic potential (80). Research has demonstrated that integrating CeO_2_ NPs into bioactive glass 3D printed scaffolds produces an anti-inflammatory therapeutic effect and promotes osteogenesis (81). This is because CeO_2_ NPs have antioxidative properties and can increase mineral deposition, alkaline phosphatase, and osteogenic gene expression. Furthermore, incorporating NPs into the scaffold can improve mechanical properties, biocompatibility, adequate cell adhesion, and osteogenic ability.

## Application and effectiveness for bone regeneration

Various applications of nanomaterials for delivering proteins in tissue regeneration therapy are summarized in Supplementary Table S1. Protein charge, characteristics, and functional groups influence trapping or incorporation in the nanocarrier and scaffold (82,83). Incorporation of BMP2 growth factor may increase the zeta potential of the heparin/polyethylene imine (PEI) nano gels, which is possibly due to the positive charge of the BMP2 protein. This is because the BMP2 protein is loaded in heparin-PEI through electrostatic interactions and binding of the N-terminal of the BMP2 molecule to heparin. The relationship between protein loading and nanocarriers was also highlighted in studies on polylactic-co-glycolic acid (PLGA) and TGF-β1 nanoparticles. PLGA nanoparticles yielded an encapsulation efficiency (EE%) of 63.93%, resulting in an actual charge concentration of approximately 1.28 ng TGF-β1 per mg nanoparticles. The zeta potential test of the PLGA-TGF β1 nanocarrier showed negative results, while the zeta potential test of the blank PLGA nanocarrier produced neutral results. This outcome indicates that loading TGF-β1 onto the PLGA nanocarrier changes its zeta potential value. TGF-β1 is known to have a negative charge because its amino acids have a negative charge (84).

The relationship between the nanocarrier surface charge and the active ingredient's charge was also examined in a study by Shen et al. (72). MSN nanocarriers with negative surface charges can be charged with positively charged drugs. In this study, bFGF with a primary isoelectric point (pI=9.6) causes the bFGF protein to have a positive charge. Furthermore, the negatively charged MSN absorbs the positively charged bFGF to be incorporated with bFGF-MSNs. Another example is the incorporation of RNA into the nanocarrier system where the RNA is loaded onto the exosome using eletrocorporation.

In a study of nano gels loaded with BMP2 (heparin/PEI-BMP2) onto a CaP ceramic scaffold, dopamine coating on the scaffold was utilized as a medium in the incorporation of the two. Dopamine contains catechol functional groups and amines, such as the DOPA side chain. DOPA side chain can be deposited as a stable layer on the surface of substrates to obtain a stable layer through oxidation reactions and spontaneous polymerization in a weak base buffer (85).

Other studies have utilized payloads for nanocarrier incorporation in scaffolds. The silica-coated scaffolds used in this study have a negative charge that can bind to positively charged BMP2, whose isoelectric point is 8.5, through electrostatic interactions. Moreover, BMP2 can bind to gelatin through hydrophobic interactions. Due to this non-covalent interaction, BMP2 was retained in the scaffold, resulting in a slow and sustained release over four weeks (86).

Polymer-based nanocarriers also utilize payloads for bonding. Because exosomes are negatively charged, cationic polymers can be added to them by LBL self-assembly to create cationic liposomes, which can then carry pDNA. PEI is a popular cationic polymeric film material that has been successfully and extensively researched as a non-viral delivery system. PLGA and other anionic polymers have been encapsulated in PEI to boost their ability to transport nucleic acids. Consequently, PEI can be thought of as a cationic polymer to improve pDNA delivery and enhance exosome transfection efficiency (87).

Spherical nanoparticles are easier to uptake and have superior biodistribution compared to other forms of nanoparticles. Research by Shen et al. (72) shows that bFGF-MSNs ±140 nm in size and with a zeta potential of ±37.0 are suitable for protein delivery during tissue regeneration because they can increase cell adhesion and biological activity.

Growth factors such as TGF-β1 are included in the large latent complex (LLC) and stored in the ECM of the bone. The goal of this approach is to inhibit protein-receptor binding and boost protein stability (88). During bone resorption, enzymes secreted by cells as matrix metalloproteases (MMPs) release latent TGF-β1 through LLC cleavage for further activation (88). The process of releasing and activating latent TGF-β1 differs in each phase during bone remodeling. Release and activation of low levels of latent TGF-β1 induce migration and differentiation of osteoblast-precursors toward mature osteoblasts at resorptive sites, as well as increased osteoblast proliferation, which also plays an anti-apoptotic role. Elevated amounts of TGF-β1 promote the synthesis of type I collagen, while suppressing osteoblast growth (84).

The occurrence of a sustained release effect and a sustained release profile depends on the constituent material. In studies with polymeric nanocarriers for TGF-β1 delivery, an initial burst release was detected within the first 24 h. This happens because the active substance is adsorbed onto the nanocarrier's surface. The mechanism of the copolymer degradation affects the release profile polymeric nanocarriers for TGF-β1 delivery (84). Shen et al. (72) reported that the mechanism of PLGA breakdown is the result of both bulk and surface diffusion, as well as bulk and surface erosion.

Water seeping into the matrix causes the polymer to hydrolyze into soluble oligomer and monomer products, which open up channels for protein diffusion and additional polymer erosion leading to the total dissolution of nanoparticles. Growth factors are released into the surrounding environment as the polymer breaks down. Furthermore, the biomolecular characteristics are crucial for drawing the aqueous phase into the matrix. In particular, the hydrophobicity of the polymers and the physicochemical and solubility characteristics of the inserted proteins are linked to release kinetics. In another investigation, in order to prevent the first release of internal bFGF and guarantee the stability of bFGF, a porous surface was used (72).

## Clinical considerations and future perspectives

Nanomaterials represent an area of drug delivery systems that is currently developing and has considerable future potential. Nanomaterials, which are characterized by their nanoscale dimensions, allow for the development of physical and chemical characteristics enabling them to improve performance and which can be utilized for various applications. In tissue regeneration therapy, nanomaterials are employed as delivery systems, improving the mechanical properties of the scaffold as well as materials that increase biological activity (89). These various properties are influenced by the nanomaterial's size, surface characteristics, and physicochemical properties (90). However, in the future application of nanomaterials, several factors must be considered, especially those related to toxicity, carcinogenicity, and teratogenicity.

The toxicity, carcinogenicity, and teratogenicity of nanomaterials are highly dependent on the administration route and exposure time. Data on the toxicity and distribution of the nanomaterials are derived from studies conducted using short-term exposure. Therefore, data on long-term toxicity are still lacking, and it is necessary to undertake further development and prevention studies regarding the long-term toxicity of nanomaterials. Another issue that needs to be considered in future development of nanomaterials is the gap between development in the laboratory and post-marketing applications. There are currently no international standards for risk assessment related to the use of nanomaterial products, including specific data requirements and testing strategies.

Various nanomaterials have been explored and developed to increase the effectiveness of bone regeneration therapy. The main requirements of excellent substitutes for bone tissues include good biocompatibility and osteogenic, osteoconductive, and osteoinductive activity. Therefore, modification techniques for size, surface properties, and physicochemical properties of nanomaterials should definitely be determined. The rapid development of nanomaterials at the bench or industrial scale must be well-adjusted with critical assessments related to their activity and toxicity to support good clinical uses, thus accelerating mass production for improving the quality of life of individuals in need of bone regeneration therapy.

## Supplementary Material

Click to view [pdf].
