# Supplementary material for: Nanomaterial application for protein delivery in bone regeneration therapy
Source: Braz J Med Biol Res. 2025 Feb 3;58:e14057. doi: 10.1590/1414-431X2024e14057 (PMC11793153; doi:10.1590/1414-431X2024e14057)
Supplement: Supplementary file 1 [file 1414-431X-bjmbr-58-e14057-suppl.pdf]

Table S1. Application and efficacy studies of proteins for bone regeneration.

Type	Active ingredient	Formulation	Administration route	Dose	Efficacy	Toxicity	Source
Liposomes	TGF-β1	Phosphatidylcholine: phosphatidylserine: cholesterol at a mass ratio of 7:2:1.	N/A	100 μg/mL	-Liposomes provided stable and more prolonged releases of up to 9 h.<br>-Alizarin Red S staining test indicated increased cell mineralization compared to control free liposome and free TGF-β1.<br>-RT-PCR test showed an increase in DMP-1 and DSPP proteins, biomarkers of dentine-pulp regeneration biomineralization.	Cell viability test shows good biocompatibility properties with cell viability values of >90%.	(5)
Microspheres	TGF-β1 dan IGF-1	Poly (alanine ethyl ester-co-glycine ethyl ester) phosphazene microspheres contained span 80 (1%, w/v), polyvinyl alcohol (1%, w/v), and Tween 60 (1%, w/v) used for loading 40 μg/ml rhTGF-β1 or 200 μg/ml rhIGF-I by double emulsion methods. PAGP microspheres solution was then dropped into dry DBM and subjected to vacuum lyophilizing to obtain 2 mg PAGP microspheres/scaffold (diameter 4 mm, height 2 mm).	Incorporation into demineralized bone matrix (DBM) scaffold and subcutaneously implanted in nude mice for eight weeks.	DBM scaffold was implanted subcutaneously in the armpit of mice at a dose of 2 mg PAGP microspheres/scaffold.	-PCR test on mice showed an increase in chondrogenic and cartilage matrix formation markers on GF microsphere-loaded DBM scaffold.<br>-GF microsphere-loaded DBM scaffold greatly increased the production of collagen and glycosaminoglycans *in vivo*.<br>-Neo-cartilage tissue used in ECM assessment of mice showed increased expression of glycosaminoglycan markers in GF scaffold microspheres.	-PCR test on BMSCs confirmed good biocompatibility properties of microsphere-incorporating DBM scaffold based on the expression of positive phenotypic markers CD44 (99.98%) and CD90 (99.60%).<br>-Neither inflammation responses nor infections were observed during the *in vivo* study.	(57)
Mesoporous Silica Nanoparticles (MSNs)	bFGF	0.4 g of cetyltrimethylammonium bromide and 2 mL of tetraethyl orthosilicate prepared by emulsion method. bFGF was loaded into MSNs by adding 45 mg MSNs to 1.5 mg/mL bFGF solution and then vacuum dried.	Local injection into the distal femur defects.	Sterilized MSNs-bFGF solution at a dose of 400 μg/L.	-Osteogenesis-related genes (RUNX2, OCN, Osterix, ALP) are upregulated.<br>-At two and four weeks of observation, radiological and histological analyses of *in vivo* bone defect repair experiments revealed enhanced bone regeneration.	-Studies of MSNs loaded bFGF on MC3T3-E1 cells viability showed good compatibility.<br>-No discernible inflammatory reaction was observed in the mice.	(72)
Adhesive Liposomes	bone morphogen-nic proteins (BMP-2)	Lecithin/cholesterol/octadecylamine (40:10:2 w/w). The liposomes were then loaded into hydrogels composed of SH-PEG and $AgNO_3$.	100 μL of lipo-hydrogel was injected into the osteoporotic fracture site and the bone marrow cavity.	20 μg BMP-2/mL.	The liposomes hydrogel significantly improved bone mineralization, fracture healing, and acceleration of osteogenesis.	Cell Counting Kit-8 (CCK-8) assay test on BMSCs cells showed that the liposomes hydrogel had good biocompatible properties.	(82)
Heparin/ Polyethylenimine (PEI) Nanogels	(BMP-2)	Heparin/polyethyleneimine (2:1, w/w).	BCP ceramics loaded heparin/PEI-BMP-2 nano-gels implanted to muscle pouches in the rectus femoris of mice (intramuscular).	5 mm BCP ceramics immersed in a solution containing 200 ng heparin/PEI-BMP-2 nanogels/mL.	-Release test showed slow release (47.84%) over 14 days.<br>-*In vitro* scratch assay test indicated an increase in cell migration after the scaffold nano-gels formulation.<br>-PCR test showed increased expression of *BMP2*, *RUNX2*, *ALP*, and *Bsp* genes in the scaffold nanogels formulation.<br>-*In vivo* test at eight weeks showed that BCP/lining/NP/BMP2 promoted bone regeneration, which almost filled all macropore scaffolds.	AlamarBlue analysis of BMSCs cultures proved that the carrier produced good cell viability.	(85)
Calcium Sulphate/Nano-hydroxyapatite-based Nano-cement	BMP-2 and bone marrow mesenchymal stromal cells derived exosomes	Nanohydroxyapatite and CSH were mixed at a weight ratio of 40:60, to produce nanocement. BMP (solution) and zoledronic acid (solution): nano cement were mixed at a ratio of liquid to powder: 600 μL/g.	Impacted into the bone defect of osteoporotic models.	BMP-2:4 μg/animal and exosomes: 10 μg/animal were added and impacted into 75 mg nanocement.	Using nanocement with BMPs and zoledronic acid induced increased bone formation with complete defect healing and enhanced biomechanical strength.	–	(61)
Thermosensitive Liposome	Parathyroid hormone-related protein	The liposome composed of DPPC, 1-mono stearoyl phosphatidylcholine	Incorporation into a collagen-hydroxy-apatite scaffold.	5 mg/mL	-The formula can provide efficiency of up to 35%.	-*In vitro* study on the MC3T3-E1 cell line showed that the	(83)

	(PTHrP 107–111)	(MSPC), 1,2-distearoyl-sn-glycero-3-phosphoethanolamine-n-[metoksi (polities glycol)-2000] (DSPE-PEG2000), and DSPE-PEG2000-maleimide at a molar ratio of 86:10:2:2. Liposome was prepared by thin film method using 5 mL of a 5 mg/mL PTHrP 107–111 solution as the hydrating solution. The liposome was then attached to the collagen-hydroxyapatite scaffold by soaking the scaffold in the liposome for thiol-maleimide interaction.			-Thermosensitive liposomes can provide slow release from lipo-scaffolds (total release of ~50% of the cargo in 13 days).<br>-With heat induction, the release increases by up to 5 times<br>-Scaffold liposomes with thermal induction produced a higher pro-osteogenic activity as indicated by an increase in ALP, OCN, and OPN gene expression and anti-osteoclastic activity as indicated by reduced levels of RANKL.	scaffold-loaded PTHrP107-111 liposomes had good cytocompatibility.<br>-Analysis of DNA contained in cells shows that scaffold liposomes are biocompatible.	
Nanocomposite Fibrous Scaffold	ONO-1301 and BMP2	Silica-coated nano-hydroxyapatite-gelatin reinforced with poly (L-lactic acid) (PLLA) fibrous yarns.	Silica coated nano-hydroxyapatite-gelatin reinforced with poly (L-lactic acid) (PLLA) fibrous yarns scaffolds were implanted into the calvarial defect sites of 4–5 months old male Wistar rats	5 µg BMP2/75 mm^3 scaffold	-BMP2 shows slow release up to day 7, followed by a linear and sustained release for 28 days.<br>-The BMP2 and ONO scaffold groups demonstrated the highest ALP activity and mineralization.<br>-*In vivo* studies showed that BMP2 and ONO scaffold groups resulted in earlier and more effective new bone formation and organization.	Studies on BMSc cells proved that the nanocomposite fibrous scaffold had good compatibility.	(86)
Exosomes	Protein within exosomes	Exosomes extracted from human adipose-derived mesenchymal stem cells (hAMSCs).	Subcutaneous implantation of exosomes silk fibroin 3D-scaffolds in nude mice	35 µg exosomes/ scaffold	-*In vitro*, differentiation tests on *ALP*, *RUNX2*, *OCN*, *COL1*, *BSP*, and *OPN* genes showed increased expression of each gene in the scaffold with exosome.<br>-*In vivo*, histological observations with H&E staining showed an increase in collagenous tissues in the exo-scaffold.<br>-MT, COL1, OPN, or RUNX2 staining showed increased expression indicating osteogenic differentiation in the scaffold-exosomes formula.<br>-The healing activities on defective bone occurred from the peripheral and central regions, resulting in bone mass density two times higher than that without an exosome-loaded scaffold during treatment lasting 5 weeks.	-The CCK-8 proliferation assay on hBMSC cells was higher in exo scaffolds than scaffolds alone at each measured time, confirming excellent biocompatibility and facilitated cell growth.<br>-There were no significant inflammatory responses or immune reactions during the *in vivo* studies.	(45)
Exosomes	DNA	Nanoparticles composed of polyethyleneimine and exosome derived from mesenchymal stem cells (5:1) were added to pDNA at a ratio of PEI nitrogen/pDNA phosphate (N/P) equal to 1:10.	Implanted in the lateral side of the defective femur of rabbits.	DBM scaffold loaded nanoparticles, including 10 µg pBMP-2.	-The ALP and alizarin red s staining test showed an increase in vitro cell differentiation in the DBM-Exosome nanoparticles.<br>-Immunohistochemical tests with OCN and OPN showed increased bone regeneration activity in the DBM-exosome nanoparticles group.	-The viability of MSCs was 97.39% for the *in vitro* study of nanoparticles assessed by MTS assay.<br>-DBM-loaded exosome nanoparticles had good biocompatibility, while without exosome, they produced high cell toxicity.	(87)
Exosomes	siRNA of the *Shn3* gene	Exosomes extracted from iPSCs and MSCs. To create exosomes modified with a bone-targeting peptide, BT-Exo-siShn3, the bone-targeting peptide modified with a diacyl-lipid tail was anchored onto the exosome.<br>About 10 µL exo (1012 particles/mL) and 90 µL of the DSPE-PEG-Mal-Cys-SDSSD (10 µM) were	IV injection once a week for 6 weeks in the tail vein of female mice.	100 µL PBS containing 1.0×10^{11} exosomes particles per mL	-BT-Exo-si*Shn3* facilitated bone formation and inhibited osteoclast formation *in vivo.*<br>-BT-Exo-si*Shn3* successfully reduced the number of bone resorption markers and increased that of bone formation markers.	-The CCK-8 assay test on cells showed the biocompatible properties of BT-Exo-si*Shn3.*<br>-No histomorphological changes were observed in the brain, heart, liver, spleen, lung, or kidney, suggesting safe use and well-tolerated BT-Exo-si*Shn3* treatment in the systemic term.	(46)

		added into 100 µL PBS (BT-Exo). A small amount of 100 µg siRNA/miRNA and 10 µL BT-Exo (1012 particles/mL) were then combined with 400 µL of cold electroporation buffer.					
PLGA Polymeric Nanocarrier	TGF-β1	PLGA and TGF-β1 50:50 to produce 1.28 ng TGF-β1 per mg of PLGA-nanoparticles (PLGA-NP). The PLGA-NP was then loaded into 1.5% type I collagen solution and fabricated into a scaffold.	Type I collagen nanostructured 3D printed loaded PLGA-NP.	100 ng/mL	-Initial burst release from PLGA-NP showed approximately 37.83 ± 2.12%, followed by a continuous release of TGF-β1 culminating in 97.57% cumulative release in 28 days.<br>-Scaffold-loaded PLGA-NP released 13.29% of TGF-β1 after 24 h of incubation, reaching up to 78.28% after 28 days.<br>-About 3.39 ± 1.15% of the scaffold area has been covered by TGF-β1, similar to the distribution area detected in bone extracellular matrix.	N/A	(84)
